# Understanding the Barriers of Implementing a Self-Awareness Assessment in Occupational Therapy Practice within a Brain Injury Population: An Exploratory Study

**DOI:** 10.1155/2023/3933995

**Published:** 2023-05-24

**Authors:** Anika Cheng, Rebecca Tsow, Julia Schmidt

**Affiliations:** ^1^Graduate Program in Occupational Therapy, University of British Columbia, Canada; ^2^Department of Occupational Science and Occupational Therapy, University of British Columbia, Canada; ^3^Rehabilitation Research Program, GF Strong Rehabilitation Centre, Canada

## Abstract

**Background:**

Self-awareness is seldom formally assessed by occupational therapists among individuals with traumatic brain injury (TBI). However, impaired self-awareness is prevalent and has a significant impact on rehabilitation outcomes. There is a need to understand clinician perspectives on self-awareness assessments and promote evidence-based practice in clinical settings.

**Aims:**

(1) Explore how an education session impacts knowledge and use of self-awareness assessments in occupational therapists working with people with TBI; (2) Understand the barriers that occupational therapists experience when assessing self-awareness in clinical practice.

**Materials and Methods:**

A single-group pre-post session design with an integrated knowledge translation approach was used. Occupational therapists working in neurorehabilitation were recruited from two rehabilitation centres through convenience sampling. Participants completed questionnaires before, after, and three months following an education session about the Self-Awareness of Deficits (SADI) assessment.

**Results:**

14 occupational therapists participated in this study. A statistically significant increase in knowledge and confidence in using the SADI was observed both post-session and at 3-month follow-up.

**Conclusion:**

Targeted and ongoing education promotes confidence and knowledge retention among occupational therapists. Further research should explore strategies to promote behaviour change. *Significance*. The barriers identified in this study can provide insights for knowledge translation across clinical contexts.

## 1. Introduction

Traumatic brain injury (TBI) has been increasingly recognized as a global health priority [1]. Common causes of moderate to severe TBI are motor vehicle accidents, falls, and assaults [2]. Importantly, TBI is the leading cause of injury-related death and disability with annual global costs of care at an estimated $400 billion [3, 4].

People with TBI can experience significant changes in function across physical, emotional, social, vocational, and cognitive domains [5–8]. Cognitive-related changes such as reduced attention, memory, processing speed, and executive function can greatly impact one's daily life, participation, and fulfillment in their home, vocational, and community roles [8–10]. Cognitive impairment can drastically disrupt one's ability to perform basic activities of daily living [11–13], such as driving, meal preparation, and money management [10].

Self-awareness is a common cognitive impairment after TBI [14, 15], which is described as one's knowledge of their abilities and limitations [16, 17]. The Dynamic Comprehensive Model of Awareness [16, 17] views self-awareness as a dynamic relationship between knowledge, beliefs, and task demands. The model defines self-awareness as having two components: (1) online awareness, which is activated during a task or situation to monitor performance, and (2) metacognitive knowledge, which is the knowledge one has prior to a task or situation [17]. In this way, while an individual may have intact metacognitive knowledge, they may require time to recognize and adjust to changes in their abilities post-injury through the use of online awareness [16, 17].

Impaired self-awareness can directly undermine rehabilitation efforts and result in negative life outcomes [18]. For example, impaired self-awareness is associated with decreases in motivation, safety in the community, and instruction comprehension [15]. Given that people with impaired self-awareness often do not recognize their limitations, they may not see a reason to engage and participate in therapy and thus may not receive the benefits and improved outcomes that these interventions would facilitate [15, 18]. As self-awareness is important for updating self-perceptions following a TBI, impaired self-awareness may prevent acknowledgement of and adaptation to changes in oneself after a TBI [19, 20].

Given the consequence and influence of self-awareness after TBI, occupational therapists have reported high importance in addressing self-awareness as a rehabilitation goal [21]. Evidence-based intervention to improve self-awareness in a supportive and constructive manner can minimise distress [22] and improve rehabilitation efficacy [15].

Assessment of self-awareness after TBI is complex [17], often conducted through comprehensive tools or measures [23]. There are various methods of assessing self-awareness including self-proxy rating discrepancy, performance-based discrepancy, structured interviews, and clinician ratings [23, 24]. Though there are many methods and types of assessments for self-awareness after TBI, only three assessments have been considered acceptable for routine and clinical research due to their psychometric and conceptual properties [23, 24]: the Self-Awareness of Deficits Interview (SADI) [25], the Patient Competency Rating Scale (PCRS) [26], and the Awareness Questionnaire (AQ) [27]. The AQ and PCRS are questionnaire-based measures, while the SADI uses a structured interview approach [23, 28, 29]. These are also the most widely used and validated scales [23, 24]. Despite their acceptability, a chart review study by Mamman et al. [30] demonstrated that formal self-awareness assessments and interventions were seldom employed by clinicians in an inpatient rehabilitation setting.

In summary, there is recognition of the impact of impaired self-awareness on rehabilitation outcomes [15], the importance of assessing self-awareness to allow clinicians to tailor interventions to improve self-awareness [31], and research indicating that occupational therapists do not employ self-awareness assessments [30, 32]. It is necessary to understand how to improve occupational therapy knowledge and the use of self-awareness assessments. Understanding the influence of an evidence-based education session for occupational therapists on the use of self-awareness assessments in clinical settings can facilitate evidence-based practice. Additionally, by understanding the barriers that clinicians experience in clinical practice, we can better target recommendations to facilitate the use of assessments.

## 2. Aims

The primary aim of this study is to explore how an education session impacts knowledge and use of self-awareness assessments among occupational therapists working with people with TBI. The secondary aim is to understand the barriers that occupational therapists experience when assessing self-awareness in clinical practice.

## 3. Materials and Methods

### 3.1. Design

This study used a single-group pre-post session design with an integrated knowledge translation approach. In integrated knowledge translation, researchers collaborate with stakeholders who provide insight throughout the research process and are impacted by the research recommendations in their own practice [33, 34]. Quantitative data were collected through questionnaires conducted via Qualtrics at three timepoints: (1) prior to the education session; (2) immediately following the education session; and (3) three months following the completion of the education session. Ethics approval for the study was obtained from the University of British Columbia's Behavioural Research Ethics Board. The data are reported using the COnsolidated criteria for REporting Qualitative research (COREQ) [35].

### 3.2. Participants

Registered occupational therapists in British Columbia were invited to participate in this study. The inclusion criteria were as follows: (1) provides rehabilitation in outpatient or inpatient clinical settings; (2) has a caseload with neurorehabilitation clients (e.g., people with stroke, traumatic brain injury, and multiple sclerosis); (3) is able to communicate in English; (4) is able and willing to participate in a 1-hour in-service session; and (5) is able to access the Qualtrics online questionnaire platform. Eligible participants were recruited by email through convenience sampling from GF Strong Rehabilitation Centre and Queen's Park Care Centre. No relationships were shared between the authors and participants prior to study commencement aside from previous research-related encounters.

### 3.3. Procedure

The SADI was selected for the education module of this study as we determined that it was the most appropriate assessment for occupational therapists to use in an inpatient rehabilitation setting. The SADI is a thorough self-awareness assessment tool that allows for setting realistic goals and anticipating the consequences of impairments, both of which are useful strategies in rehabilitation [23]. Due to its adaptable format [28], the SADI may also be better suited for the conversational nature of most occupational therapy sessions.

An education module was developed and structured based on the BOPPPS model of lesson planning, which includes a bridge, learning objectives, pre-assessment, participatory learning session, post-assessment, and summary [36]. This model was selected for its emphasis on engaging participant interest, assessing understanding as a session progresses, and reinforcing key ideas at the end of the session [36, 37].

The education module was delivered in-person by two female undergraduate research assistants with training in neurological rehabilitation (authors AC and RT). Participants were informed of the research team's context, level of experience, and goal to increase use of self-awareness assessments with this study. The in-service was 1-hour long and consisted of the following components: (1) *introduction and learning objectives*; (2) *overview of self-awareness and formal self-awareness assessments*; (3) *evidence for formal self-awareness assessments in client outcomes*; (4) *how to use the SADI*; and (5) *interactive practice using the SADI*. In addition, a summary booklet was developed to complement module content, which was emailed to all participants after the in-service and presented in hard copy for occupational therapist coordinators to keep onsite at both rehabilitation facilities.

### 3.4. Data Collection Procedures

An online questionnaire was developed to capture participant use of and perceptions about formal self-awareness assessments throughout the study. Participants were asked to complete the online questionnaire at three timepoints: prior to the education session, immediately following the session, and three months after completion of the session, as was done in similar studies [37]. This timing of data collection allowed us to observe both immediate and lasting changes in participant behaviour and perceptions following education. Demographic information such as age, sex, employment status, workplace setting, TBI caseload, and years of experience were also collected to characterize the participants in the study.

The questionnaire consisted of three main components. Section one included one question about the current use of formal self-awareness assessments that were only administered at pre-session and follow-up timepoints. Participants selected a response worded in the first person, ranging from “none” to “all” with respect to the number of clients they had used SA assessments with. Section two captured participant perceptions about formal self-awareness assessments under four domains: (1) *current engagement*; (2) *knowledge and confidence*; (3) *preferences and treatment styles;* and (4) *beliefs about efficacy and importance.*Section 3 was used to investigate a fifth domain, *barriers,* through qualitative prompts. Domains were developed from the theoretical domain framework, a validated integration of theories which describes 14 domains including knowledge, skills, beliefs about capabilities, environmental context and resources, and social influences [38]. The theoretical domain framework enables researchers to identify, understand, and address behaviour change in knowledge translation research [39].

Specific questionnaire items were informed by relevant, published tools assessing clinician perspectives [40, 41] as well as theories of behaviour change [42]. Theories were used to guide the inclusion and wording of questionnaire items; for example, since participant motivation is important for the behaviour change process [42], the item “I would rather not use formal self-awareness assessments” was included on the questionnaire. Items within the *knowledge and confidence* domain were adapted from an educational module by Roy et al. [37] exploring physiotherapists' perceptions of mirror therapy. Finally, prior to its implementation in this study, the questionnaire was piloted by an occupational therapist partner who provided feedback and made revisions to improve the relevance of the questionnaire for occupational therapists.

The first four domains in section 2 involved three Likert-scale (5-point) questions each for a total of 12 questions, and the barriers domain included one multiselect and one open-ended question. The open-ended question was used to capture facilitators of performing formal self-awareness assessments in clinical practice that might have been missed by quantitative items. Finally, questionnaire wording was guided by the BRUSO model, which recommends that items be brief, relevant, unambiguous, specific, and objective for greatest questionnaire efficacy [43].

### 3.5. Statistical Analysis

Quantitative data were analysed using SPSS Statistics. Demographic data were reported using frequencies and percentages. Responses to the 12 5-point Likert-scale items were coded as scores, where a score of one represented statements such as “never” and “strongly disagree,” and a score of five represented statements such as “always” and “strongly agree”. Higher scores represented beliefs or practices that favoured the use of formal self-awareness assessments. All item response scores within each domain (current engagement, knowledge and confidence, preference and treatment style, and beliefs about efficacy and importance) were averaged and compared across the three timepoints with descriptive statistics such as mean and standard deviation. The open-ended question was analysed using summative content analysis [44] to report the frequency of key ideas mentioned.

Section one of the questionnaire focused on clinicians' current use of formal self-awareness assessments. Ratings of the use of self-awareness assessments at pre-session and 3-month follow-up timepoints were collected. Clinicians were not asked to rate their use of assessments at the post-session timepoint as the post-session questionnaire was administered immediately following the education session. Results were analysed using descriptive statistics given that no statistical tests were appropriate for the small sample size and zero baseline values of our data. In section two of the questionnaire, participants described their perceptions about formal self-awareness assessments; this section of the questionnaire was provided at pre-session, post-session, and follow-up timepoints. Questions were related to current engagement with self-awareness questionnaires, knowledge and confidence, preference and treatment style, and beliefs about their efficacy and importance. These data were analysed with a repeated measures ANOVA, setting statistical significance at *p* < 0.05. Section three of the questionnaire asked about general barriers to self-awareness assessment use through a selection of priority choices. These responses were analysed through frequencies and percentages. In section four of the questionnaire, participants were asked to describe their reasons for not using formal self-awareness assessments regularly and provide any additional comments in an open-ended format. Responses were categorised based on common themes for descriptive purposes.

## 4. Results

A total of 14 occupational therapists were recruited across two sites for this study, with seven participants from each site. Demographic characteristics are shown in Table 1. Almost all participants were full-time occupational therapists with at least one year of occupational therapy experience. At the time of recruitment, the majority of participants indicated that moderate-to-severe TBI cases comprised less than 50% of their caseloads. All participants completed pre-session, post-session, and follow-up questionnaires (i.e., no missing data). The average use of assessments at pre-session and follow-up timepoints is described in section 4.1 of the results.  Table 2 reports the averages of each domain at each timepoint (pre-session, post-session, and follow-up) and Table 3 reports the comparisons of each domain at each timepoint (pre-session, post-session, and follow-up). The results below indicate participant responses within the five domains of our questionnaire, as developed from the theoretical domain framework.

### 4.1. Current Use of Formal Self-Awareness Assessments

A 14% increase (SE = 0.097; SD = 0.363; variance = 0.132) in the use of formal self-awareness assessments was observed between pre-session and 3-month follow-up. At baseline, there were no participants who had used formal SA assessments with any of their clients in the past three months. At the time of follow-up, two of the 14 participants had begun using formal self-awareness assessments with up to 50% of their clients since the education session.

### 4.2. Perceptions of Self-Awareness Assessment Use

**Figure 1:**
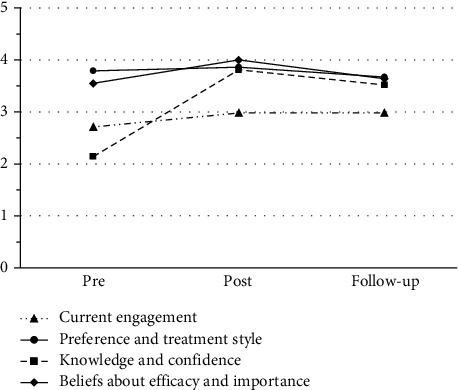
Perceptions about formal assessments.

All participants (*N* = 14) described their perceptions related to SA assessment use under the four domains: current engagement, knowledge and confidence, preference and treatment styles, and beliefs about efficacy and importance (Supplemental Table 1). The following describes each domain, the findings, and the statistical analysis used to determine significance across timepoints (Table 2, Figure 1, and Table 3).

The current engagement domain was not statistically significant; however, there was an increasing trend towards engagement and use of self-awareness assessments from pre- to post-session timepoints which was maintained at follow-up (*F*(2, 82) = 2.804; *p* = 0.07).

The domain of knowledge and confidence showed a significant increase across two timepoints (*F*(2, 82) = 51.360; *p* = <0.001). The most significant increases were between pre- and post-session timepoints and between pre-session and follow-up timepoints. While there was a decrease in average score between post-session and follow-up timepoints, it remained higher than at pre-session levels. Post hoc pairwise comparisons using the Bonferroni correction showed increases between pre- and post-session (*p* = <0.001) and pre-session and follow-up (*p* = <0.001), and a decrease between post-session and follow-up (*p* = 0.037) timepoints, where the difference between the first two timepoints was statistically significant.

For the domain of preference and treatment styles, there was an increase in mean scores between pre- and post-session timepoints; however, at follow-up, the increase dropped below pre-session levels. All three timepoints were not statistically significant and remained relatively constant (*F*(2, 82) = 0.923; *p* = 0.40).

For the beliefs about efficacy and importance domain, mean scores between pre- and post-session timepoints increased and were statistically significant (*F*(2, 82) = 6.860; *p* = 0.002). The difference between pre-session and follow-up was not statistically significant (*p* = 1.000); however, the follow-up score was higher than the pre-session score. Post hoc pairwise comparisons using the Bonferroni correction determined that there was a significant increase between pre- and post-session timepoints (*p* = 0.004), however there was also a significant decrease between post-session and follow-up timepoints (*p* = 0.008).

Figure 1 depicts changes from pre-session (before the education session), post-session (immediately after the education session), and follow-up timepoints (three months after the education session). The graph shows the biggest change in the knowledge and confidence domain, with the greatest increase from pre- to post-session timepoints. The other three domains of current engagement, preference and treatment styles, and beliefs about efficacy and importance demonstrated an increase from pre- to post-session but remained relatively stable or decreased at follow-up. By the 3-month follow-up, the majority of increases from post-session had decreased.

### 4.3. Barriers

Participants reported a range of barriers in our study. Prior to the education session, most participants (*N* = 13, 92.9%) indicated that they were not familiar with self-awareness assessments. However, following the education session, only one participant (7.1%) at post-session and no participants at 3-month follow-up reported unfamiliarity with self-awareness assessments. Similarly, many participants felt under-equipped to use formal self-awareness assessments in practice prior to the education session (*N* = 11, 78.6%), but only three participants (21.4%) reported the same after the education session and at 3-month follow-up. Far more participants indicated that they did not feel limited by barriers to formally assessing self-awareness at follow-up (*N* = 7, 50.0%) than prior to (*N* = 0) or immediately following the session (*N* = 2, 14.3%). Precise multi-select response frequencies can be found in Supplemental Table 2.

### 4.4. Additional Comments

Open-ended responses were also collected about barriers to regularly using self-awareness assessments. Lack of familiarity was the top-reported barrier among comments gathered pre-session (*N* = 10, 71.4%). One participant shared, *“I am not aware of any formal assessments related to this—in my practice, it has always been targeted informally through assessment [of] other cognitive components or functional skills. However, I could very much see the benefit of utilising this measure”* (P1). Immediately following the session, a prior lack of awareness (*N* = 7, 53.8%) and a willingness to try using formal self-awareness assessments (*N* = 5, 38.4%) were the most common ideas referenced in participant comments. At the time of follow-up, no participants reported a lack of familiarity with self-awareness assessments. Instead, lack of preparation or confidence in administering the tests (*N* = 4, 33.3%) or a preference for assessing self-awareness informally (*N* = 4, 33.3%) were the most commonly listed reasons for not using self-awareness assessments regularly. For example, one participant expressed that they *“often use[d] the SADI questions, but “informally”, i.e., asking the questions but not completing the full paper/pencil assessment”*(P2). Three participants (25.0%) described already using informal self-awareness assessments in their practice at baseline. At follow-up, four participants (33.3%) discussed using informal assessments, two of whom had not mentioned doing so before the education session.

## 5. Discussion

This study is one of the first to explore occupational therapists' use of formal self-awareness assessments before and after an education session using an integrated knowledge translation approach. Clinician attitudes towards and engagement with self-awareness assessment tools significantly improved immediately following the education session; most notable was an increase in occupational therapist knowledge and confidence, persisting even three months afterward. Barriers identified in this study can provide future insights for promoting behaviour change among clinicians in healthcare and rehabilitation contexts.

Our findings indicate that barriers related to knowledge gaps can be successfully addressed through education [37]. Unfamiliarity was the most prevalent barrier to using self-awareness assessments, with improvements in knowledge and confidence demonstrated after an education session. Notably, knowledge levels remained improved even three months after the education session. Understanding the barriers that impact occupational therapy practice can provide guidance for developing specific and targeted strategies to mitigate these barriers [45, 46]. Providing education and training on methods of measuring self-awareness may also improve the clinical use of assessments.

Our education session employed key participatory design research methods described by VanHeerwaarden et al. [47] which may increase participant uptake of knowledge. For example, our education sessions incorporated small group discussions and facilitated participant confidence through acknowledgement of their expertise. As identified by Pashmdarfard et al. [48], clinical education is crucial for linking theoretical courses to practice and professionalism. Other studies have also found education sessions to be effective in clinical settings when they are interactive, centred on the values of stakeholders, and incorporate participatory design methods (37). Roy et al. [37] had similar success with providing an education session about mirror therapy to physiotherapists. In our study, engagement strategies such as structuring education sessions with the BOPPPS model may have boosted the efficacy of knowledge dissemination.

Our study supports existing literature to suggest that even a single in-service can increase knowledge and reinforce evidence-based practice. However, clinician preference and treatment styles did not change after the education session. Clinicians in our study recognized self-awareness as a high priority, even from baseline. Similarly, Winkens et al. [32] found that while a majority (71%) of occupational therapists considered self-awareness important for rehabilitation, only 7% used standardised assessments that were specific to self-awareness. This reflects the discrepancy between what occupational therapists believe is important and how they conduct clinical practice. In other literature, participants also acknowledged the importance of self-awareness [21, 49], yet there was a difference between how occupational therapists intended to use self-awareness assessments and how they actually used them [50]. These findings reveal that there are complex and multifaceted factors influencing clinical use of self-awareness assessments, such as one's clinical judgement, reasoning, and practice [50]. While changing established routines in occupational therapy practice is difficult, targeting specific factors such as familiarity with assessments, time to complete assessments, work environment, and work culture can impact clinician's choice and use of assessments [50].

Our participants had varied lengths of work experience as occupational therapists, which may have influenced our data. Five out of 14 occupational therapists had less than two years of experience, four had between three and 10 years of experience, and five had over 10 years of experience. Newer occupational therapists tend to choose practical, shorter, and more familiar assessments [50], and thus may be more comfortable using assessments and interventions that they already know. This may have influenced our findings; participants with fewer years of work experience may not feel as comfortable exploring new assessments and may need more time to integrate new assessments into their daily practice.

One key factor influencing behaviour change is a person's motivation [42]. Motivation is particularly important for learning and retaining knowledge and skills [51]. Previous research indicates that increased engagement and acceptance of new assessments and treatment styles can be developed through clinicians' involvement in knowledge translation interventions [39]. Our study attempted to increase participant motivation to engage in the education session through participatory action research methods, such as including input from an occupational therapist partner when designing the study and employing interactive discussions throughout the education session.

The literature on the connections between knowledge, confidence, and practice is mixed. While some studies have found that increases in clinician confidence [52] or beliefs [37] did not lead to meaningful changes, others have identified a lack of knowledge and confidence as the main barriers to implementing knowledge into practice [39]. For example, Zieber and Sedgwick [51] observed increased participant ratings of competence, knowledge, and confidence both immediately and three months after a training activity with nursing students. Their findings suggest that increased confidence enhances competence and knowledge retention, thereby promoting more effective learning. In our study, participants maintained knowledge and confidence in self-awareness assessments even three months after the education session, with a general non-significant decline compared with immediately after the session. The average use of self-awareness assessments also increased following the education session. Though this change was not statistically significant, it was observed despite no reminders, check-ins, or other interventions from the research team during the 3-month window prompting assessment use or refreshing clinician's knowledge.

Increasing knowledge retention may promote evidence-based practice. In this study, significant improvements in clinician's engagement and beliefs about the efficacy and importance of self-awareness assessments were observed post-session but not maintained at follow-up. Knowledge and confidence ratings also declined from post-session to follow-up. During the 3-month window between the two timepoints, occupational therapists may have forgotten some of their new beliefs, convictions, and knowledge from the education session. Since knowledge level affects both confidence and competence [51], it is worth exploring whether greater behaviour changes could be observed by increasing clinician exposure to session material. This could include disseminating infographics with key concepts, asking the occupational therapist coordinator to remind clinicians where assessment sheets are located, or directly contacting individual clinicians to ask about their progress using self-awareness assessments [53]. Based on their research on the effects of different pedagogical methods on long-term retention, Baker and Robinson [54] suggested increasing the duration of content “soak time” by introducing new concepts slowly, testing knowledge repeatedly over a period of time, and providing retrieval cues to remind individuals of what they have learned.

Overall, our findings indicate that targeted, ongoing education about formal self-awareness assessments is important for clinician retention of knowledge, which may then translate to behaviour change.

## 6. Limitations

There are two main limitations to this study relating to sampling and bias. We used convenience sampling techniques to recruit eligible participants, which may have captured an inaccurate representation of the general clinician population resulting in low levels of reliability and high levels of bias. Moreover, due to our small sample size, only descriptive statistics were provided about clinician use of self-awareness assessments in our study. In addition, low power due to our small sample size could have resulted in the lack of significance in our results. Future research should further investigate changes in assessment use after an education session through statistical analysis. Our small sample size may have also resulted in low generalizability to the wider population of occupational therapists. However, this sample size was comparable to previous studies using similar methods [37, 50] and included participants with diverse ages and experience levels in brain injury rehabilitation.

Response bias may have affected the results of this study. Since our education module emphasised the importance of formally assessing self-awareness, participants may have responded in ways that they perceived as favourable to our research at the post-session and follow-up timepoints. Furthermore, participants from both rehabilitation facilities were familiar with members of the research team through prior research and clinical practice activities, which may have also affected their participation in our study. Throughout the data collection process, participants were reassured that their responses would not be linked to their identities, which may have helped to mitigate the impacts of response bias.

## 7. Significance

This study explored how an education session impacts the knowledge and use of self-awareness assessments by occupational therapists working with people with TBI. The role of formal self-awareness measurement tools in facilitating rehabilitation outcomes has been demonstrated in numerous studies. Based on this study and others' findings, clinician attitudes and behaviours largely influence their practices in conducting self-awareness assessments. Providing education is effective in improving clinician knowledge and confidence in using formal self-awareness assessments, and even one education session can lead to increased knowledge uptake in practice. Though this study only observed a small increase in use of self-awareness assessments, providing education shows promise for prompting behaviour changes in clinicians. Further investigation of the most appropriate and effective knowledge translation activities for clinicians would be helpful to understand the types, methods, and timing of education that enables knowledge translation in clinical settings. Future research should consider persisting barriers to implementation identified in this study and explore strategies to promote the use of formal self-awareness assessments in clinical practice.

## Figures and Tables

**Table 1:** Demographics.

Age distribution	*N* (%)
25-34	7 (50.0)
35-44	5 (35.7)
45-54	—
55-64	2 (14.3)
Employment status	
Full-time (>35 hrs/week)	13 (92.9)
Part-time (<30 hrs/week)	1 (7.1)
Workplace setting	
Inpatient	8 (57.1)
Outpatient	5 (35.7)
Acute	1 (7.1)
Caseload with moderate to severe TBI	
0-24%	7 (50.0)
25-49%	5 (35.7)
50-74%	—
75-100%	2 (14.3)
Years of experience	
Less than 1 year	1 (7.1)
1-2 years	4 (28.6)
3-5 years	3 (21.4)
6-10 years	1 (7.1)
10+ years	5 (35.7)

**Table 2:** Average domain scores.

	Pre Mean (SD)	Post Mean (SD)	Follow-up Mean (SD)
Current engagement	2.71 (1.24)	2.98 (1.20)	2.98 (1.16)
Knowledge and confidence	2.14 (1.03)	3.81 (0.74)	3.52 (0.89)
Preference and treatment style	3.79 (0.92)	3.86 (0.75)	3.67 (0.79)
Beliefs about efficacy and importance	3.55 (0.86)	4.00 (0.73)	3.64 (0.88)

*N* = 14. Scores are out of five with five meaning “strongly agree”.

**Table 3:** Post hoc pairwise comparison.

	Time points		Mean difference (SE)	*p* value
Current engagement	Pre	Post	-0.262 (0.113)	0.078
	Pre	Follow-up	-0.262 (0.128)	0.141
	Post	Follow-up	0.000 (01.41)	1.000

Knowledge and confidence	Pre	Post	-1.667 (0.204)	<0.001^∗^
	Pre	Follow-up	-1.381 (0.199)	<0.001^∗^
	Post	Follow-up	0.286 (0.109)	0.037

Preferences and treatment style	Pre	Post	-0.071 (0.150)	1.000
	Pre	Follow-up	0.119 (0.141)	1.000
	Post	Follow-up	0.190 (0.133)	0.479

Beliefs about efficacy and importance	Pre	Post	-0.452 (0.133)	0.004^∗^
	Pre	Follow-up	-0.095 (0.140)	1.000
	Post	Follow-up	0.357 (0.112)	0.008^∗^

*N* = 14; ^∗^significant at *p* < 0.05; SE: standard error.

## Data Availability

The data generated and analysed during the study are available from the authors on request.

## Supplementary Materials

Supplementary Materials Supplemental Table 1 includes the questions provided on the pre-session, post-session, and follow-up questionnaires which were reported on in section 4.2 of the results. Three questions relating to perceptions of self-awareness assessments were posed for each of the four domains and respective responses were scored from “strongly agree” (5) to “strongly disagree” (1). Supplemental Table 2 lists all multi-select response frequencies reported in section 4.3 of the results. Participants were asked to select as many responses as they identified with. Rows correspond to response options provided on the questionnaire. Data from pre-session, post-session, and 3-month follow-up timepoints are presented as frequency counts and percentages in the right-hand columns.
