# Supplementary material for: Understanding the Barriers of Implementing a Self-Awareness Assessment in Occupational Therapy Practice within a Brain Injury Population: An Exploratory Study
Source: Occup Ther Int. 2023 May 24;2023:3933995. doi: 10.1155/2023/3933995 (PMC10232193; doi:10.1155/2023/3933995)
Supplement: Supplementary Materials — Supplemental Table 1 includes the questions provided on the pre-session, post-session, and follow-up questionnaires which were reported on in section 4.2 of the results. Three questions relating to perceptions of self-awareness assessments were posed for each of the four domains and respective responses were scored from “strongly agree” (5) to “strongly disagree” (1). Supplemental Table 2 lists all multi-select response frequencies reported in section 4.3 of the results. Participants were asked to select as many responses as they identified with. Rows correspond to response options provided on the questionnaire. Data from pre-session, post-session, and 3-month follow-up timepoints are presented as frequency counts and percentages in the right-hand columns. [file 3933995.f1.docx]

**Supplemental Table 1.** Questions about Perceptions of Self-Awareness Assessments

| **Domain** | **Questions** |
| --- | --- |
| **Current Engagement** | I observe decreased self-awareness in my clients |
|  | I have opportunities to learn about new assessments and implement them if I want to |
|  | I use formal self-awareness assessments (i.e., SADI, PCRS, AQ) in regular practice |
| **Knowledge and Confidence** | I assess self-awareness appropriately with my clients |
|  | I am familiar with the formal self-awareness assessments (i.e., SADI, PCRS, AQ) that we currently use in practice |
|  | I feel confident in using formal self-awareness assessments with my clients |
| **Preferences and Treatment Style** | I would rather not use formal self-awareness assessments |
|  | I prioritise formally assessing other issues (e.g., ADLs, RTW, memory) over self-awareness when treating clients |
|  | Understanding my client’s level of self-awareness impacts my decision making |
| **Beliefs about Efficacy and Importance** | I believe formal self-awareness assessments are important for client rehabilitation outcomes |
|  | I find formal self-awareness assessments more useful than informal self-awareness assessments |
|  | The usefulness/importance of formal self-awareness assessments depends on each client |

**Supplemental** **Table 2.** Barriers

|  | **Pre-test**  n (%) | **Post-test**  n (%) | **Follow-up**  n (%) |
| --- | --- | --- | --- |
| I am not familiar with the formal self-awareness assessments available | 13 (92.9%) | 1 (7.1%) | 0 |
| I feel under-equipped to use formal self-awareness assessments | 11 (78.6%) | 1 (7.1%) | 3 (21.4%) |
| Formal self-awareness assessments are time-consuming | 6 (42.9%) | 2 (14.3%) | 6 (42.9%) |
| Formal self-awareness assessment results make my clients upset | 1 (7.1%) | 1 (7.1%) | 0 |
| I do not formally assess SA | 8 (57.1%) | 8 (57.1%) | 4 (28.6%) |
| I do not feel limited by barriers to formally assessing SA | 0 | 2 (14.3%) | 7 (50.0%) |
| Other | 0 | 2 (14.3%) | 7 (50.0%) |
